# Effects of Microencapsulated and Nanoemulsified β-Carotene on Antioxidant and Anti-Inflammatory Function in Weaned Piglets

**DOI:** 10.3390/antiox15081046

**Published:** 2026-08-21

**Authors:** Qi Zhu, Fudong Zhang, Zhu Zhu, Xinyuan Ye, Ge Zhang, Zeyu Zhang, Jinbiao Zhao

**Affiliations:** State Key Laboratory of Animal Nutrition and Feeding, College of Animal Science and Technology, China Agricultural University, Beijing 100193, China; s20243040844@cau.edu.cn (Q.Z.);

**Keywords:** β-carotene, microencapsulated formulation, nanoemulsion formulation, antioxidation, anti-inflammation

## Abstract

β-carotene has excellent antioxidant and anti-inflammatory activities. However, its poor chemical stability and susceptibility to oxidative degradation strongly limit its practical application. Therefore, encapsulation technology serves as a crucial delivery method to achieve the bioavailability of β-carotene. The study aimed to explore effects of microencapsulated and nanoemulsified β-carotene on antioxidant and anti-inflammatory function in weaned piglets. A total of 147 healthy weaned piglets with similar initial body weight (7.75 ± 0.09 kg) were randomly divided into three groups: control group (CON), microencapsulated β-carotene treatment group (B1) and nanoemulsified β-carotene treatment group (B2). Results showed that the two β-carotene preparations remarkably improved growth performance, antioxidant capacity and immunity of weaned piglets compared with the CON group (*p* < 0.05). In tissues of liver, jejunum and colon, B1 and B2 groups increased antioxidase activity (CAT, SOD, T-AOC) and concentrations of anti-inflammatory cytokines (IL-2, IL-4, IL-10, IFN-γ), with reduced MDA and pro-inflammatory cytokines (IL-1β, IL-6, IL-12, TNF-α) contents. Both B1 and B2 treatments improved intestinal barrier function and optimized jejunal microbial composition compared with the CON group (*p* < 0.05). The B2 group exerted far superior antioxidant and anti-inflammatory effects compared with the B1 group (*p* < 0.001). In conclusion, the nanoemulsion delivery system can improve antioxidant and anti-inflammatory function of β-carotene in weaned piglets compared with the traditional microencapsulated form. Our findings provide a theoretical basis for the popularization and application of nanoemulsified β-carotene to improve the bioavailability of β-carotene.

## 1. Introduction

β-Carotene is a highly bioactive lipophilic carotenoid with potent antioxidant and anti-inflammatory physiological functions [1]. Its molecular structure is abundant in conjugated double bonds, which can efficiently scavenge excess free radicals in the organism and alleviate oxidative stress. Meanwhile, it can suppress excessive inflammatory responses, regulate the secretion of inflammatory cytokines, and relieve inflammatory injuries [2]. Therefore, β-carotene plays a critical role in alleviating oxidative damage and maintaining systemic immune homeostasis. Nevertheless, abundant conjugated double bonds result in poor chemical stability of β-carotene [3]. Light, high temperature, acid–base conditions and the oxidative environment of the gastrointestinal tract all trigger the oxidative degradation of β-carotene [4]. In addition, β-carotene has weak water solubility, resulting in poor dispersibility and low in vivo bioavailability [5].

Encapsulation-based delivery technology is an indispensable technical method for its industrial application. Spray-dried microcapsules are currently the most mature and widely commercialized encapsulation system for β-carotene production. In this process, high-molecular polymers such as starch, maltodextrin and whey protein isolate are adopted as wall materials to encapsulate the core material β-carotene, and solid microcapsule particles are fabricated via high-pressure spray drying [6]. The microcapsule structure can partially isolate β-carotene from oxidative surroundings and improve its stability during processing and storage [7]. However, conventional microcapsules exhibit limitations in β-carotene loading [8]. Moreover, relatively large oil-core droplets are released after microcapsules are hydrated in aqueous gastrointestinal fluids. These droplets cannot readily penetrate the intestinal mucus barrier [9], ultimately leading to limited absorption and utilization efficiency. In contrast, nanocapsules are smaller than the pores of the intestinal mucus layer. They can carry encapsulated β-carotene to pass through the mucus layer intact, and then be directly absorbed by intestinal epithelial cells via multiple cellular transport pathways, featuring a shorter absorption pathway and remarkably enhanced absorption efficiency [10]. Compared with solid microencapsulation systems, liquid emulsion delivery systems contain tiny oil droplets that can fully contact lipase to promote lipid hydrolysis and accelerate the incorporation of β-carotene into mixed micelles, thereby presenting superior bioaccessibility [11]. With the continuous iterative optimization of delivery carrier technology, nanoemulsion encapsulation techniques have become increasingly sophisticated. With the assistance of emulsifiers, the processing system disperses oil-phase β-carotene into nanoscale droplets, which combine favorable water dispersibility and high loading capacity for lipophilic active substances. Nano-sized droplets rapidly penetrate the intestinal mucus layer, shorten the release cycle of active ingredients, and markedly increase the intestinal accumulation and absorption of β-carotene in the host intestine [12].

Theoretically, nanoemulsions can compensate for the shortcomings of traditional microcapsules, including slow core release and insufficient bioavailability. However, current comparative studies on different encapsulated β-carotene formulations are mostly limited to in vitro physicochemical characterization and simulated digestion assays, lacking in vivo experimental evidence under real physiological digestive conditions [13]. Weaned piglets are susceptible to oxidative damage and inflammatory responses owing to their immature immune system, which leads to poor growth performance. We hypothesized that nanoemulsified β-carotene would exert superior effects on improving growth performance and antioxidase activities compared with microencapsulated β-carotene. Therefore, this study was carried out to explore the effects of microencapsulated and nanoemulsified β-carotene on the growth performance, antioxidant capacity and immune function in weaned piglets.

## 2. Materials and Methods

### 2.1. Materials

Microencapsulated β-carotene (5% purity) and nanoemulsified β-carotene (5% purity) were purchased from Shandong Haineng Bioengineering Co., Ltd. (Rizhao, China). The two β-carotene delivery systems in this study were processed from the same whey-protein-containing primary emulsion. The primary emulsion was subjected to multi-step emulsification to produce liquid nanoemulsion, which was used as nanoemulsion-formulated β-carotene for group B2. The resulting nanoemulsion was thoroughly mixed with maltodextrin serving as wall material and then spray-dried to obtain microencapsulated β-carotene powder for group B1. Kits for the detection of digestive enzyme activities, intestinal barrier function markers, antioxidase activity and inflammatory cytokines were obtained from Nanjing Jiancheng Bioengineering Institute (Nanjing, China). The high-definition constant H&E staining kit was purchased from Servicebio Technology Co., Ltd. (Wuhan, China).

### 2.2. Determination of Physicochemical Properties of Microencapsulated and Nanoemulsified β-Carotene Under Storage Conditions

#### 2.2.1. Droplet Size Analysis

The particle size distributions of β-carotene nanoemulsions and dispersed microencapsulated suspensions were determined using a dynamic light-scattering instrument (Nanotrac Wave, Microtrac, Inc., Montgomeryville, PA, USA). The microencapsulated powder was gently stirred in deionized water to obtain a homogeneous suspension before further dilution. Prior to measurement, both the microencapsulated suspension and nanoemulsion samples were diluted 100-fold with deionized water to achieve an identical β-carotene concentration for the two systems. The storage stability of microencapsulated and nanoemulsified β-carotene delivery systems was evaluated by the periodic particle-size determination at 5-day intervals over a 60-day storage period. Both delivery systems were stored at two temperatures (4 °C and 25 °C) to comparatively explore the effect of the storage temperature on particle-size changes during long-term preservation. All measurements were performed in triplicate.

#### 2.2.2. Turbidity Measurement

The spectrophotometric method was adopted to evaluate the particle aggregation behavior of two β-carotene delivery systems: β-carotene microcapsule suspension (B1) and β-carotene nanoemulsion (B2). Turbidity was measured at 600 nm with a UV–Vis spectrophotometer (Model S-220, Boeco, Hamberg, Germany), according to the protocol reported by Mehmood and Ahmed [14].

Prior to testing, B1 microcapsule powder was dispersed in deionized water under gentle stirring to form homogeneous suspension. The loading amounts of β-carotene and olive oil were kept identical across the two groups to eliminate concentration interference. B1 and B2 samples were separately stored at 4 °C and 25 °C for 30 days, and the turbidity was detected at 5-day intervals to record the turbidity variation induced by droplet aggregation during storage. All measurements were performed in triplicate.

#### 2.2.3. Total Color Difference

The non-destructive colorimetric method reported previously was adopted to evaluate the color fading degree of two β-carotene delivery systems: β-carotene microcapsule suspension (B1) and β-carotene nanoemulsion (B2) [15].

Prior to detection, B1 microcapsule powder was dispersed in deionized water under gentle stirring to form a homogeneous suspension. The contents of β-carotene and olive oil were kept identical between the two groups to eliminate concentration interference. Samples of B1 and B2 were stored separately at 25 °C and 55 °C for 30 days, and the total color difference (TCD) was measured at 5-day intervals.

An appropriate volume of each sample was transferred into a transparent colorimetric cuvette. The tristimulus color parameters L*, a* and b* were recorded by a portable colorimeter (DR900, HACH, Dubai, United Arab Emirates), where L* stands for lightness, a* for red–green axis value and b* for yellow–blue axis value. The total color difference (TCD) was calculated using the following formula:

$$TCD={\left[{\left(L_{1}^{*}-L_{0}^{*}\right)}^{2}+{\left(a_{1}^{*}-a_{0}^{*}\right)}^{2}+{\left(b_{1}^{*}-b_{0}^{*}\right)}^{2}\right]}^{1/2}$$

In the equation, $L_{1}^{*}$, $a_{1}^{*}$, and $b_{1}^{*}$ represent the color parameters after a designated storage period; $L_{0}^{*}$, $a_{0}^{*}$, and $b_{0}^{*}$ denote the initial color coordinates of fresh samples right after preparation. All color measurements were performed in triplicate.

#### 2.2.4. *p*-Anisidine Value Measure

The *p*-anisidine value was measured to characterize the secondary oxidative stability of olive oil within different β-carotene delivery systems. Three sample groups were prepared for comparative analysis: free olive oil mixed with β-carotene (CON, control group without encapsulating barrier), β-carotene microcapsule powder (B1), and β-carotene nanoemulsions (B2).

The loading amounts of β-carotene and olive oil were identical across all three groups to eliminate concentration interference. For the CON control, β-carotene was fully dissolved in olive oil in advance to prepare the oil mixture before fabricating B1 microcapsules and B2 nanoemulsions. Prior to accelerated storage testing, B1 microcapsule powder was dispersed in deionized water under gentle stirring to form a homogeneous suspension, ensuring uniform aqueous testing conditions for all treatments. A total of 15 g fresh sample of CON mixture, B1 suspension and B2 nanoemulsion were incubated at 50 °C for 0–7 days. At predetermined time points, 1 g sample was collected and extracted with HPLC-grade n-hexane to recover the lipid phase.

The absorbance of n-hexane extracts was first detected at 350 nm by a UV–Vis spectrophotometer and recorded as *A*_NB_ (absorbance before derivatization). The *p*-anisidine reagent was prepared by fully dissolving 2.5 g *p*-anisidine in 1 L glacial acetic acid. Afterwards, 5 mL oil extract was blended with 1 mL *p*-anisidine reagent and incubated in darkness for 10 min to complete chromogenic reaction, and the post-reaction absorbance *A*_NA_ was measured at 350 nm. Refined pure olive oil extract was adopted as blank reference.

The *p*-anisidine value was calculated via the following equation reported by Mehmood et al. [16]. All measurements were performed in triplicate, and the data were expressed as the mean ± standard deviation:

$$p-\mathrm{anisidine value}=25\times \left(1.2A_{NA}-A_{NB}\right)/M$$

where *A*_NA_ = absorbance after reaction; *A*_NB_ = absorbance before reaction; *M* = mass of tested sample (g).

### 2.3. Animal Feeding Trial

#### 2.3.1. Ethics Statement

The animal feeding trial was carried out at the Fengning Experimental Station of China Agricultural University and the Fengning Animal Experiment Base of National Feed Engineering Technology Research Center (Chengde, China). All animal experimental procedures were strictly implemented in accordance with the national standard Guidelines for Ethical Review of Laboratory Animal Welfare. Throughout the whole experimental period, piglets were raised in well-ventilated and clean standardized pens with free access to feed and fresh water. The health status of piglets was monitored daily, with feed intake and diarrhea incidence recorded correspondingly. Conventional biosecurity operations including vaccination and manure disposal were routinely performed during the trial.

#### 2.3.2. Experimental Design

A total of 147 weaned piglets at 24 days of age with an average initial body weight of 7.75 ± 0.09 kg were randomly allocated into three groups for a 28-day feeding trial, with 7 replicate pens per group and 7 piglets in each pen. Piglets in the CON group were fed a corn–soybean basal diet. For the B1 group, 5 kg of spray-dried microencapsulated β-carotene (5% active ingredient) was supplemented per ton of basal diet. The microencapsulated powder was first subjected to stepwise dry premixing with a small portion of basal feed and then thoroughly mixed with the remaining basal diet. For the B2 group, 5 kg of nanoemulsified β-carotene (5% active ingredient) was added per ton of basal diet; the nanoemulsion was diluted with a small amount of clean water and then uniformly blended into the basal diet under continuous stirring. This supplementation scheme ensured that the dietary concentration of pure β-carotene was uniformly maintained at 0.25 kg/t across all treatment groups. All experimental diets were freshly prepared and provided to piglets on the preparation day to avoid deterioration caused by moisture. The basal diet was mash feed. The ingredient compositions of the experimental diets for each group are presented in Table 1.

#### 2.3.3. Sample Collection and Analysis

All growth performance parameters were calculated based on piglet body weight recorded on day 0 and day 28 of the feeding trial, as well as the residual feed weight collected at the end of the 28-day experimental period. Piglets were fasted for 24 h before blood collection. In total, 63 blood samples were collected from the anterior vena cava of piglets using 10 mL vacuum blood collection tubes on days 0, 14 and 28 of the trial. The blood samples were centrifuged at 3500× *g* and 4 °C for 10 min to separate the plasma and serum, which were separately stored at −20 °C for subsequent biochemical analysis. For the determination of plasma retinol concentration, approximately 0.5 mL plasma was aliquoted into a 15 mL amber centrifuge tube and mixed with 3 mL of n-hexane. After 10 min of vortex mixing, the supernatant was harvested. The extraction procedure was performed in triplicate; the obtained supernatants were pooled and evaporated to dryness under a stream of nitrogen gas at ambient temperature. The dried residue was reconstituted in 0.2 mL of methanol, vortexed, and filtered through a 0.22 μm filter prior to quantification by high-performance liquid chromatography (HPLC) (HPLC, 1260 Infinity, Agilent Technologies, Santa Clara, CA, USA) equipped with a spectrophotometric detector (Agilent Technologies, USA). The assays for intestinal barrier-related biomarkers in serum were strictly conducted in accordance with the manufacturer’s instructions of the corresponding commercial kits (Nanjing Jiancheng Bioengineering Institute, China).

On day 28 of the trial, seven piglets from each treatment group were randomly chosen to obtain tissue and digesta samples. At the end of the 28-day feeding trial, piglets were humanely euthanized via intravenous injection of sodium pentobarbital (100 mg/kg body weight) prior to tissue sampling. Segments of the duodenum, jejunum and ileum were excised and fixed in 4% paraformaldehyde for 48 h. Hematoxylin–eosin (H&E) staining was carried out using a high-definition constant HE staining kit (Servicebio Technology Co., Ltd.), following the manufacturer’s instructions. For each intestinal segment per pig, three histological sections were prepared. Target tissue regions were imaged under an Eclipse Ci-L light microscope (Nikon, Tokyo, Japan) at 40× magnification. Quantitative analysis was implemented uniformly via Image-Pro Plus 6.0 software (Media Cybernetics, Silver Spring, MD, USA) with millimeters as the unit of measurement. For each histological slice, five intact villi were measured for their villus height and five crypts for their crypt depth, and the corresponding mean values were calculated. Liver, jejunal and colonic tissues were collected for subsequent molecular biological assays, including redox-status indicators (CAT, SOD, MDA, T-AOC) and immune-related cytokines (IL-1β, IL-2, IL-4, IL-6, IL-10, IL-12, TNF-α, INF-γ). Jejunal digesta samples were collected for the determination of digestive enzyme activities (amylase (AMS) and lipase (LPL)). All fresh samples, excluding tissues for fixation, were immediately snap-frozen in liquid nitrogen for further analyses. All these measurements were strictly performed according to the protocols provided by the commercial assay kits (Nanjing Jiancheng Bioengineering Institute, China).

For gut microbiota sequencing analysis, all jejunal digesta samples were transported to the laboratory on dry ice immediately after sampling to preserve the integrity of the microbial community structure. The total microbial genomic DNA was extracted using the FastPure Stool DNA Isolation Kit (MJYH, Shanghai, China). A NanoDrop 2000 micro-spectrophotometer (Thermo Fisher Scientific, Wilmington, DE, USA) was utilized to determine DNA concentration and purity, and agarose gel electrophoresis was performed to verify the integrity of DNA fragments [17].

The V3–V4 hypervariable region of the bacterial 16S rRNA gene was amplified with the primer pair 338F (ACTCCTACGGGAGGCAGCAG) and 806R (GGACTACHVGGGTWTCTAAT). After PCR amplification, target bands were verified on 2% agarose gels. The amplified products were purified with a domestic Yuhua PCR Clean-Up Kit (Shanghai Meiji Yuhua Biomedical Technology Co., Ltd., Shanghai, China) and quantified using a Qubit 4.0 fluorometer (Thermo Fisher Scientific, Waltham, MA, USA). Sequencing libraries were constructed with the NEXTFLEX Rapid DNA-Seq Kit (Revvity, Waltham, MA, USA), and paired-end 300 bp (PE300) sequencing was carried out on the Illumina NextSeq 2000 platform (Illumina, San Diego, CA, USA). After demultiplexing, the resulting sequences were quality filtered with fastp (0.19.6) and merged with FLASH (v1.2.11). High-quality sequences were processed via the DADA2 plugin embedded in QIIME 2 (2023.9) software with default parameters to generate the amplicon sequence variant (ASV) feature table [18].

### 2.4. Statistical Analysis

Differences among three experimental groups (CON, B1, B2) for all parameters except intestinal microbiota were evaluated by one-way analysis of variance (one-way ANOVA), followed by Tukey’s multiple-comparison test using IBM SPSS Statistics v22.0. All statistical graphs were visualized by OriginPro 2024 (OriginLab Corporation, Northampton, MA, USA) based on the ANOVA results. Data in figures are presented as the mean ± standard error of the mean (SEM). Table data are given as the mean ± standard deviation (SD); statistical significance was set at *p* < 0.05.

All bioinformatic analyses for intestinal microbiota were conducted on the Majorbio Cloud online platform (https://cloud.majorbio.com (accessed on 12 March 2026)). Based on the obtained ASV feature table, Mothur software (version 1.30.2) was used to compute three alpha-diversity estimators: Chao index, Ace index and Shannon index. Principal coordinate analysis (PCoA) based on the Bray–Curtis distance was applied to assess the similarity of microbial community structures across different samples, and the ANOSIM test was used to examine the statistical significance of inter-group clustering differences. The above two analyses were realized via the vegan package (v2.5-3) within R language. Differences in microbial communities among CON, B1 and B2 groups were assessed using the Kruskal–Wallis H test.

## 3. Results

### 3.1. Changes in Physicochemical Properties of Microencapsulated β-Carotene and Nanoemulsified β-Carotene During Storage

**Figure 1:**
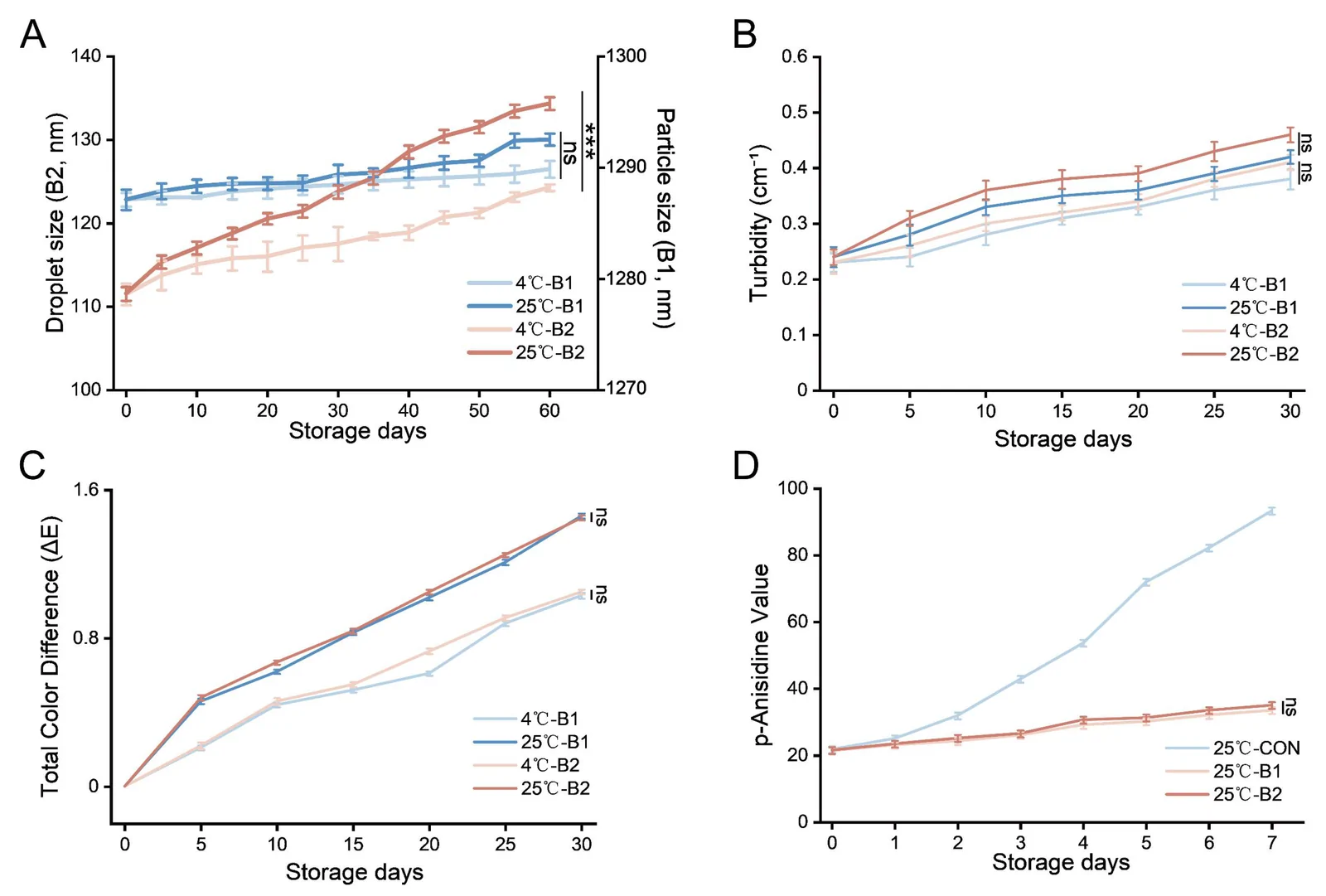
Storage characteristics of different β-carotene delivery systems. (**A**) Droplet size, (**B**) turbidity, (**C**) total color difference (ΔE) of β-carotene microcapsules (B1) and nanoemulsified β-carotene (B2) stored at 4 °C and 25 °C; (**D**) *p*-anisidine value of CON, B1 and B2 under accelerated storage at 25 °C. B1, microencapsulated β-carotene delivery system; B2, nanoemulsified β-carotene delivery system; CON, free β-carotene dissolved in olive oil. Asterisks (***): significant difference between corresponding groups; ns, no significant difference. *n* = 3. Line charts show the time-dependent variation of measured parameters throughout storage.

Figure 1A illustrates the changes in droplet size of β-carotene delivery systems during storage. It should be noted that B1 (microcapsules, particle-size range: 1280–1300 nm) and B2 (nanoemulsions, particle-size range: 110–140 nm) are displayed on separate y-axes; so, their absolute particle-size values are not directly comparable. Over the 60-day storage period, B1 exhibited only slight particle-size increase, and nearly overlapping particle-size profiles were obtained for samples stored at 4 °C and 25 °C. In contrast, B2 (nanoemulsions) showed strong temperature-dependent particle growth. For B2, the droplet size increased moderately at 4 °C, while a sharp rise was observed at 25 °C; at day 60, the droplet size of B2 stored at 25 °C was significantly higher than that of the 4 °C counterpart. These results demonstrate that elevated temperature greatly promoted the droplet aggregation in the nanoemulsion system, whereas the microencapsulated formulation offered good resistance to temperature-triggered particle enlargement. It should be noted that the particle size determined by dynamic light scattering does not represent the dimension of dry microcapsule granules but the size of β-carotene-loaded oil droplets released after microcapsule hydration. This droplet size could approximate the initial state of the oil droplets released when microcapsules encounter aqueous conditions in vivo.

Consistent trends were found for the turbidity (Figure 1B) and total color difference ΔE (Figure 1C). The turbidity and ΔE of B1 and B2 continuously rose during storage at both temperatures. No significant differences were observed between B1 and B2 under the same storage temperature for turbidity and color change. The *p*-anisidine value (Figure 1D) was applied to evaluate lipid oxidation. Under accelerated storage at 25 °C, the CON group (unencapsulated β-carotene dissolved in olive oil) displayed a sharp rise in *p*-anisidine value, representing severe lipid oxidation. In contrast, B1 and B2 showed markedly slower accumulation of oxidation products. No significant difference existed between B1 and B2, revealing that both encapsulation systems effectively retarded the lipid oxidation and possessed comparable antioxidative protection performance.

### 3.2. Effects of β-Carotene Forms on Plasma Retinol Concentration in Weaned Piglets

**Figure 2:**
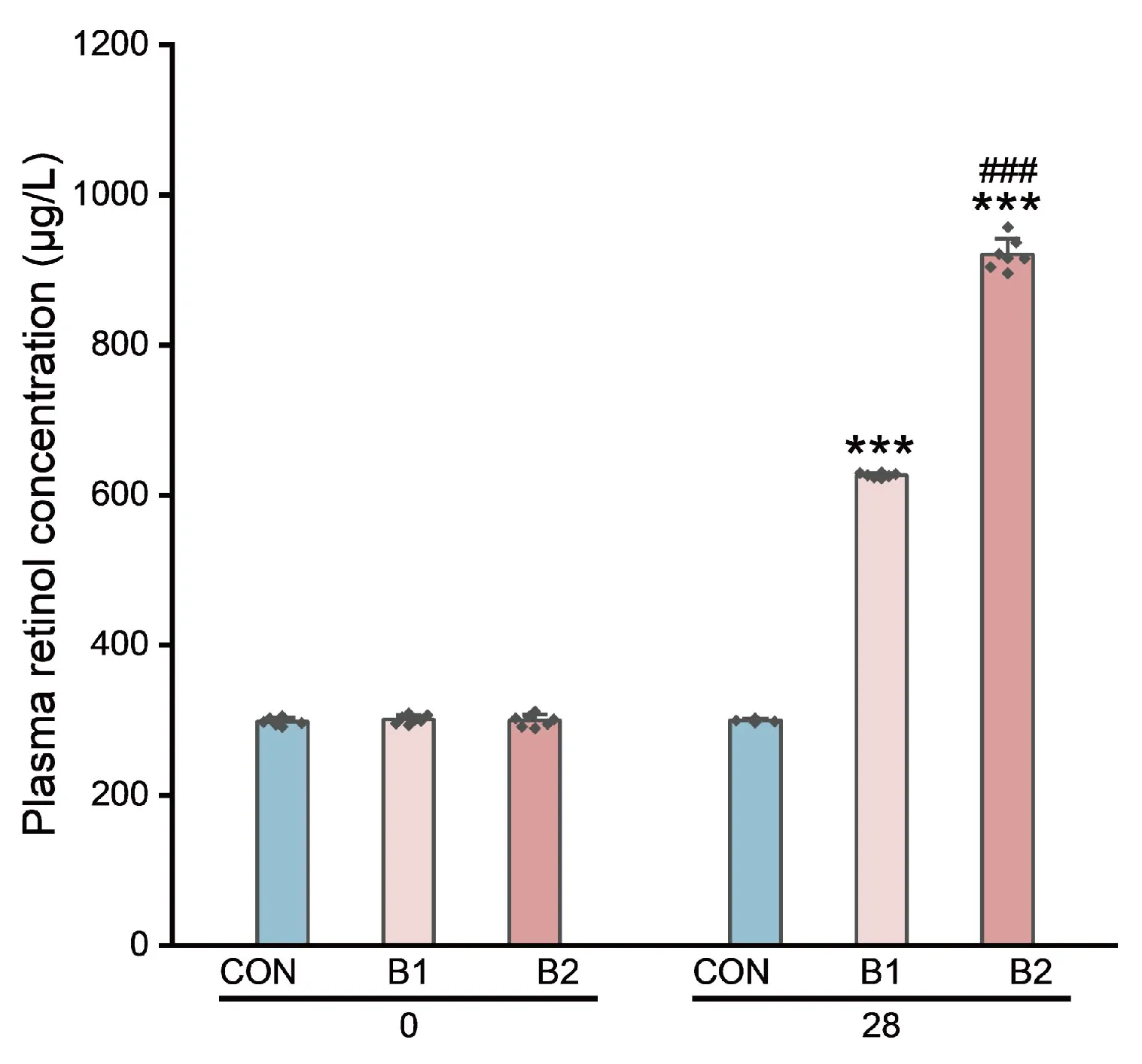
Plasma retinol concentration at day 0 and day 28 in different experimental groups. CON, basal diet control group without β-carotene; B1, basal diet supplemented with microencapsulated β-carotene; B2, basal diet supplemented with nanoemulsified β-carotene. Asterisks (***): significant difference compared with the CON group at *p* < 0.001. Hash symbols (###): significant difference between B1 and B2 groups at *p* < 0.001. *n* = 7. Data are expressed as mean ± standard error, and scattered diamond points represent individual replicate values.

As shown in Figure 2, at day 0, the plasma retinol levels showed no obvious differences among the CON, B1 and B2 groups. At day 28, the plasma retinol concentration was significantly elevated in the B1 and B2 groups relative to the CON group (*p* < 0.001); moreover, the B2 group exhibited a markedly higher plasma retinol level than the B1 group (*p* < 0.001).

### 3.3. Effects of β-Carotene Forms on Growth Performance in Weaned Piglets

The effects of dietary supplementation with microencapsulated β-carotene (B1 group) and nanoemulsified β-carotene (B2 group) on piglet growth performance are summarized in Table 2. The average daily gain (ADG) of group B1 was significantly higher than that of the CON control group (*p* < 0.05); ADG was further significantly increased in group B2 compared with group B1 (*p* < 0.05), and the average daily feed intake exhibited a similar trend. Although both B1 and B2 showed a significantly higher ADG and average daily feed intake relative to CON, no significant differences in the feed-to-gain ratio were observed between treatment groups and the control. The calculated daily β-carotene intake of CON, B1 and B2 piglets was 14.83, 319.88 and 337.70 mg/d, respectively. Notably, the dietary supplemented dosage of β-carotene was identical for B1 and B2, and the delivery form of β-carotene served as the sole experimental variable.

### 3.4. Effects of β-Carotene Forms on Activity of Amylase and Lipase

**Figure 3:**
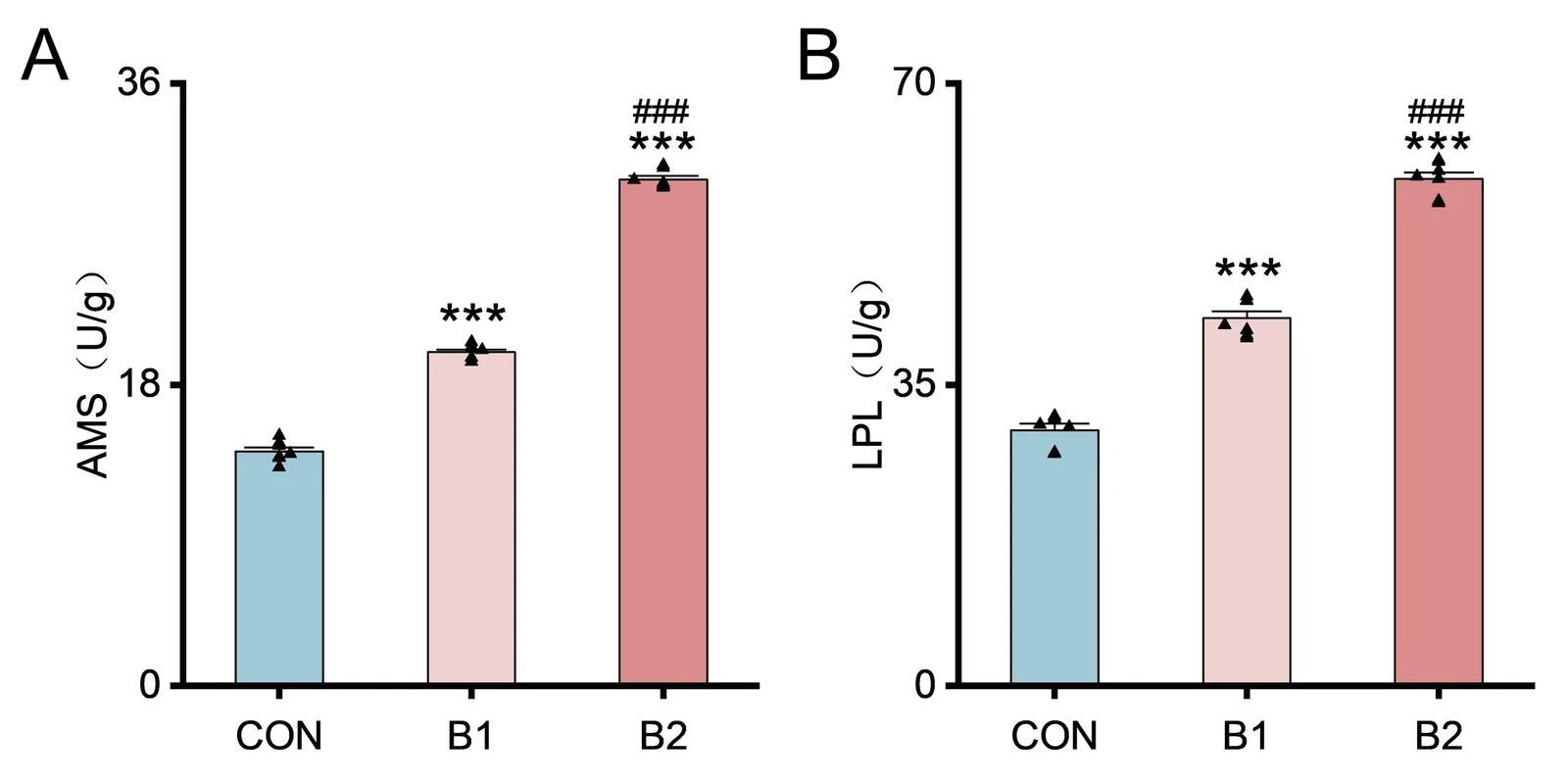
Digestive enzyme activities in jejunal tissue. (**A**) Amylase (AMS) activity of different experimental groups; (**B**) lipase (LPL) activity of different experimental groups. CON, basal diet control group without β-carotene; B1, basal diet supplemented with microencapsulated β-carotene; B2, basal diet supplemented with nanoemulsified β-carotene. Asterisks (***): significant difference compared with the CON group at *p* < 0.001. Hash symbols (###): significant difference between B1 and B2 groups at *p* < 0.001. *n* = 7. Data are expressed as mean ± standard error, and scattered triangular points represent individual replicate values.

To further elucidate the intrinsic mechanism by which different delivery forms of β-carotene alter the growth performance of piglets, we measured the activities of jejunal amylase and lipase to assess intestinal digestive function. As shown in Figure 3, the jejunal AMS and LPL activities were significantly increased in the B1 and B2 groups compared with the CON group (*p* < 0.001); furthermore, the activities of both digestive enzymes were markedly higher in the B2 group than in the B1 group (*p* < 0.001).

### 3.5. Effects of β-Carotene Forms on Hepatic Antioxidant Status and Inflammatory Cytokines

**Figure 4:**
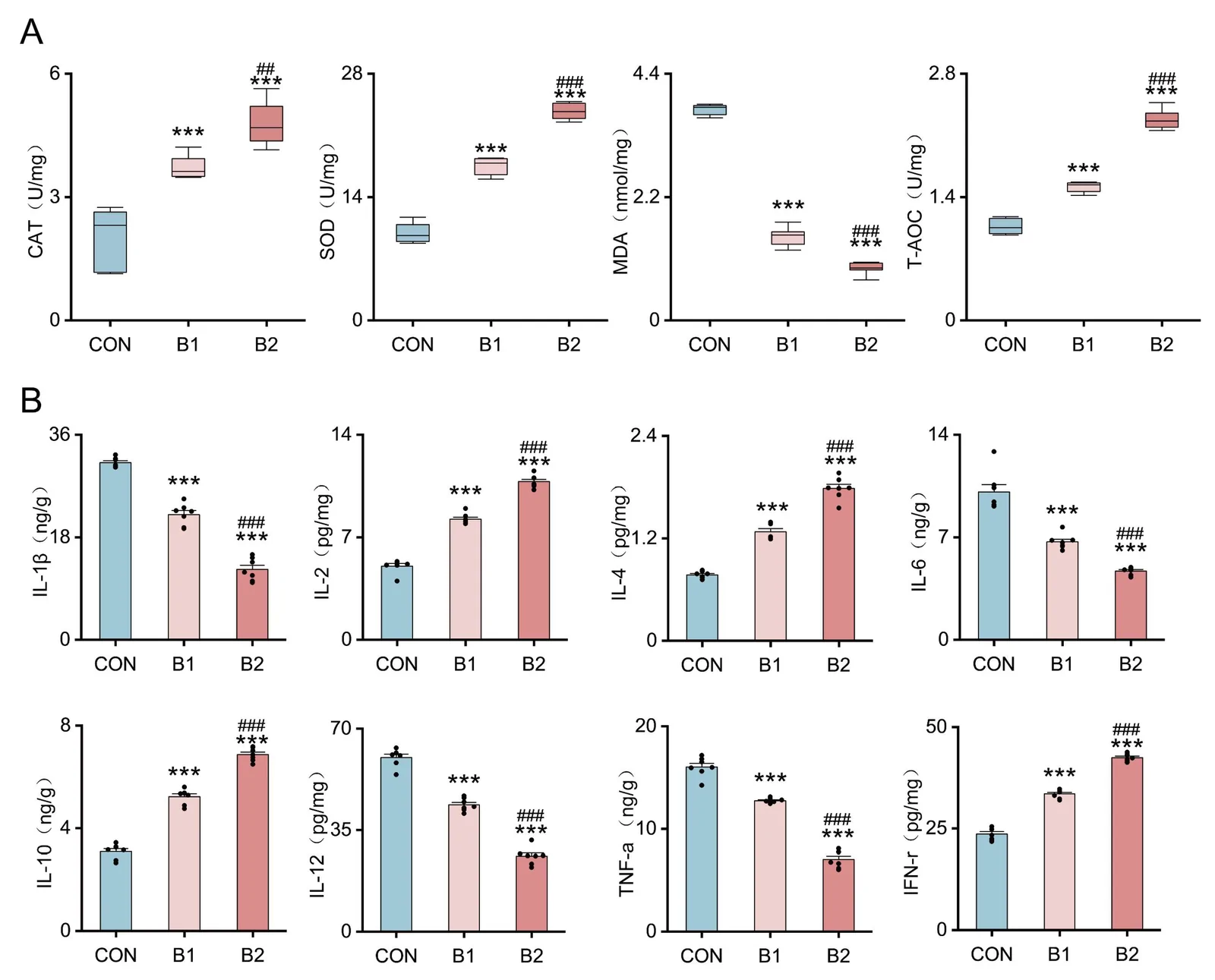
Hepatic antioxidant capacity and inflammatory cytokine levels. (**A**) Hepatic antioxidant-related indicators: catalase (CAT), superoxide dismutase (SOD), malondialdehyde (MDA), and total antioxidant capacity (T-AOC). (**B**) Hepatic inflammatory cytokine concentrations, including pro-inflammatory cytokines (IL-1β, IL-6, IL-12, TNF-α) and anti-inflammatory cytokines (IL-4, IL-10), as well as immune regulatory factors (IL-2, IFN-γ). CON, basal diet control group without β-carotene; B1, basal diet supplemented with microencapsulated β-carotene; B2, basal diet supplemented with nanoemulsified β-carotene. Asterisks (***): significant difference compared with the CON group at *p* < 0.001. Double hash symbols (##): significant difference between B1 and B2 groups at significant difference at *p* < 0.01. Hash symbols (###): significant difference between B1 and B2 groups at *p* < 0.001. *n* = 7. Box plots and bar charts display the distribution of individual replicate values.

Figure 4A exhibited the hepatic antioxidant and immune-related indicators of piglets supplemented with microencapsulated β-carotene (B1) and nanoemulsified β-carotene (B2). The results for hepatic antioxidant status showed that both the B1 and B2 groups significantly increased the activities of hepatic catalase (CAT), superoxide dismutase (SOD) and total antioxidant capacity (T-AOC), while significantly reducing the hepatic malondialdehyde (MDA) concentration compared with the CON group (*p* < 0.001). Pairwise comparison between the B1 and B2 groups revealed that B2 exerted stronger hepatic antioxidant effects: the hepatic levels of CAT, SOD and T-AOC were significantly higher, whereas MDA content was significantly lower in B2 than in B1 (*p* < 0.001), indicating that nanoemulsified β-carotene exhibits superior effects in improving the hepatic antioxidant and anti-inflammatory status of weaned piglets compared with microencapsulated β-carotene.

The hepatic inflammatory cytokines exhibited a consistent trend with the antioxidant indicators (Figure 4B). Compared with the CON group, both the B1 and B2 groups significantly downregulated the expression of pro-inflammatory cytokines IL-1β, IL-6, IL-12 and TNF-α, while significantly upregulating the anti-inflammatory cytokines IL-4 and IL-10, as well as the immune regulatory factors IL-2 and IFN-γ (*p* < 0.001). Significant differences were observed in all immune indicators between the B1 and B2 groups, and the B2 group possessed a more balanced anti-inflammatory homeostasis of hepatic cytokines (*p* < 0.001). Collectively, the combined data of hepatic antioxidant and inflammatory markers demonstrate that nanoemulsified β-carotene exerts stronger protective effects on piglet liver than microencapsulated β-carotene.

### 3.6. Effects of β-Carotene Forms on Intestinal Morphological Structures in Piglets

**Figure 5:**
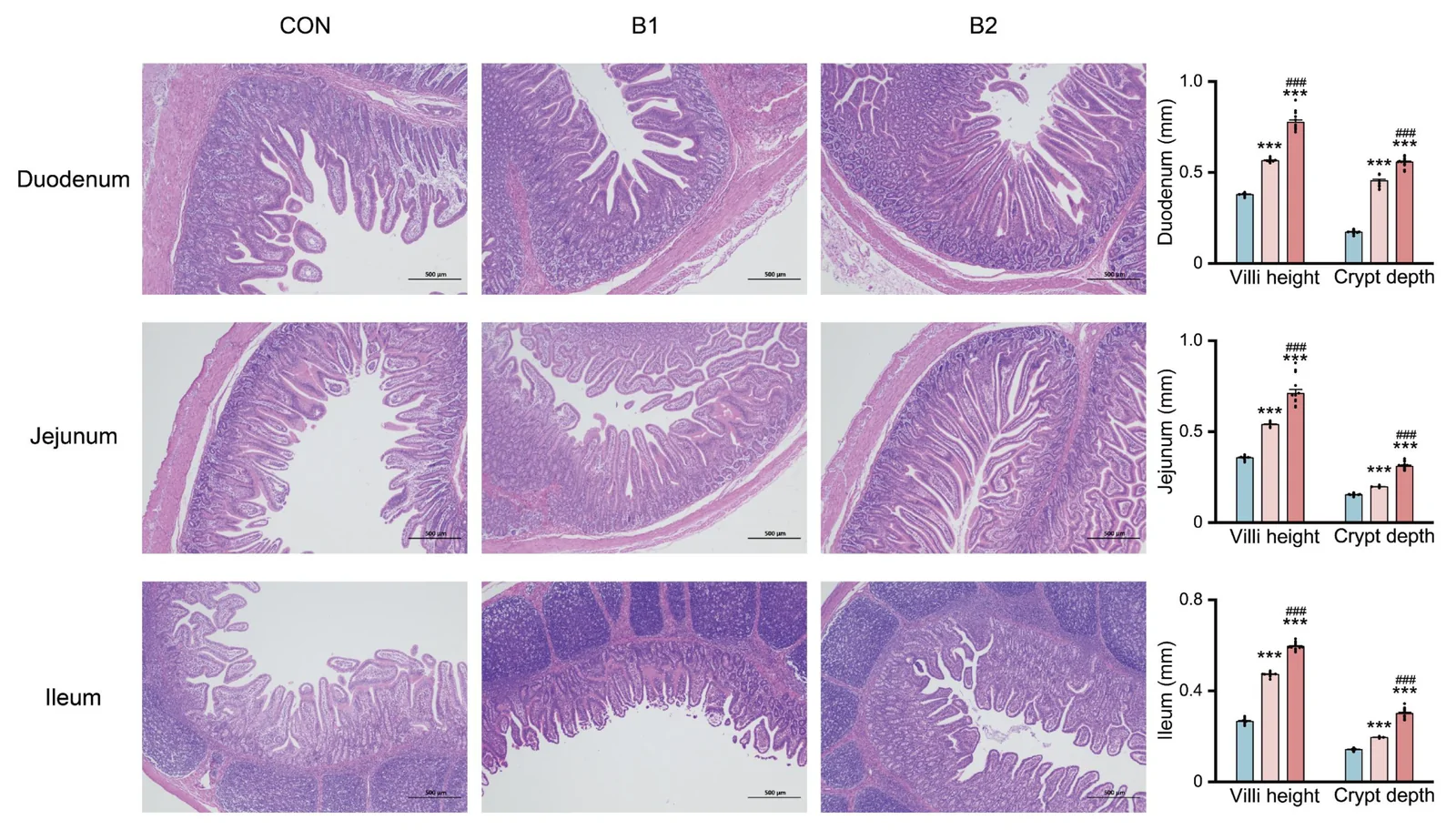
Intestinal morphological structure and morphological indices of different experimental groups. Representative hematoxylin–eosin (H&E) staining micrographs of duodenum, jejunum and ileum tissues from CON, B1 and B2 groups are shown on the left side; the corresponding quantitative statistical results of villus height and crypt depth are displayed in the bar charts on the right. Scale bar = 500 μm for all histology images. CON, basal diet control group without β-carotene; B1, basal diet supplemented with microencapsulated β-carotene; B2, basal diet supplemented with nanoemulsified β-carotene. Asterisks (***): significant difference compared with the CON group at *p* < 0.001. Hash symbols (###): significant difference between B1 and B2 groups at *p* < 0.001. *n* = 7. Data are presented as mean ± standard error, and scattered dots represent individual replicate values.

Hematoxylin–eosin (H&E) staining and quantitative analysis of duodenal, jejunal and ileal tissue sections from piglets in each treatment group showed that dietary supplementation with microencapsulated β-carotene (B1) or nanoemulsified β-carotene (B2) significantly improved the intestinal morphology and increased villus height and crypt depth (*p* < 0.001; Figure 5). Nanoemulsified β-carotene (B2) exhibited an extremely superior repairing effect on the intestinal villus–crypt structure compared with microencapsulated β-carotene (B1) (*p* < 0.001; Figure 5).

### 3.7. Effects of β-Carotene Forms on Intestinal Permeability in Weaned Piglets

**Figure 6:**
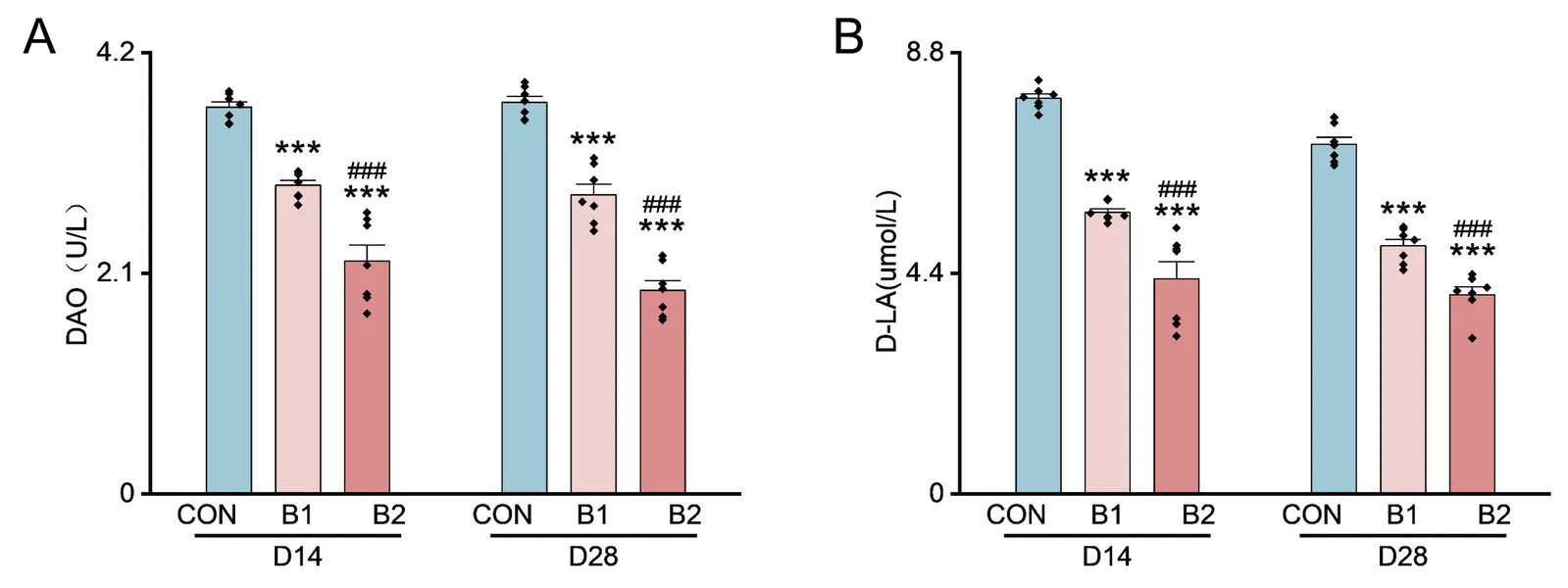
Serum intestinal barrier permeability markers on day 14 and day 28 of the trial. (**A**) Diamine oxidase (DAO) activity of different experimental groups; (**B**) D-lactic acid (D-LA) concentration of different experimental groups. Samples were collected on day 14 (D14) and day 28 (D28). CON, basal diet control group without β-carotene; B1, basal diet supplemented with microencapsulated β-carotene; B2, basal diet supplemented with nanoemulsified β-carotene. Asterisks (***): significant difference compared with the CON group at *p* < 0.001. Hash symbols (###): significant difference between B1 and B2 groups at *p* < 0.001. *n* = 7. Data are presented as mean ± standard error, and scattered dots represent individual replicate values.

Blood samples of piglets from all experimental groups were collected on day 14 and day 28 of the feeding trial to determine the serum DAO and D-LA concentrations. As illustrated in Figure 6, dietary supplementation with both delivery forms of β-carotene remarkably alleviated intestinal barrier leakage. At both sampling time points, the serum DAO and D-LA contents in the B1 and B2 groups were significantly reduced relative to the CON group (*p* < 0.001). Moreover, the B2 group exhibited much lower serum DAO and D-LA levels than the B1 group on d 14–28 (*p* < 0.001).

### 3.8. Effects of β-Carotene Forms on Jejunal Antioxidant Status and Inflammatory Cytokines

**Figure 7:**
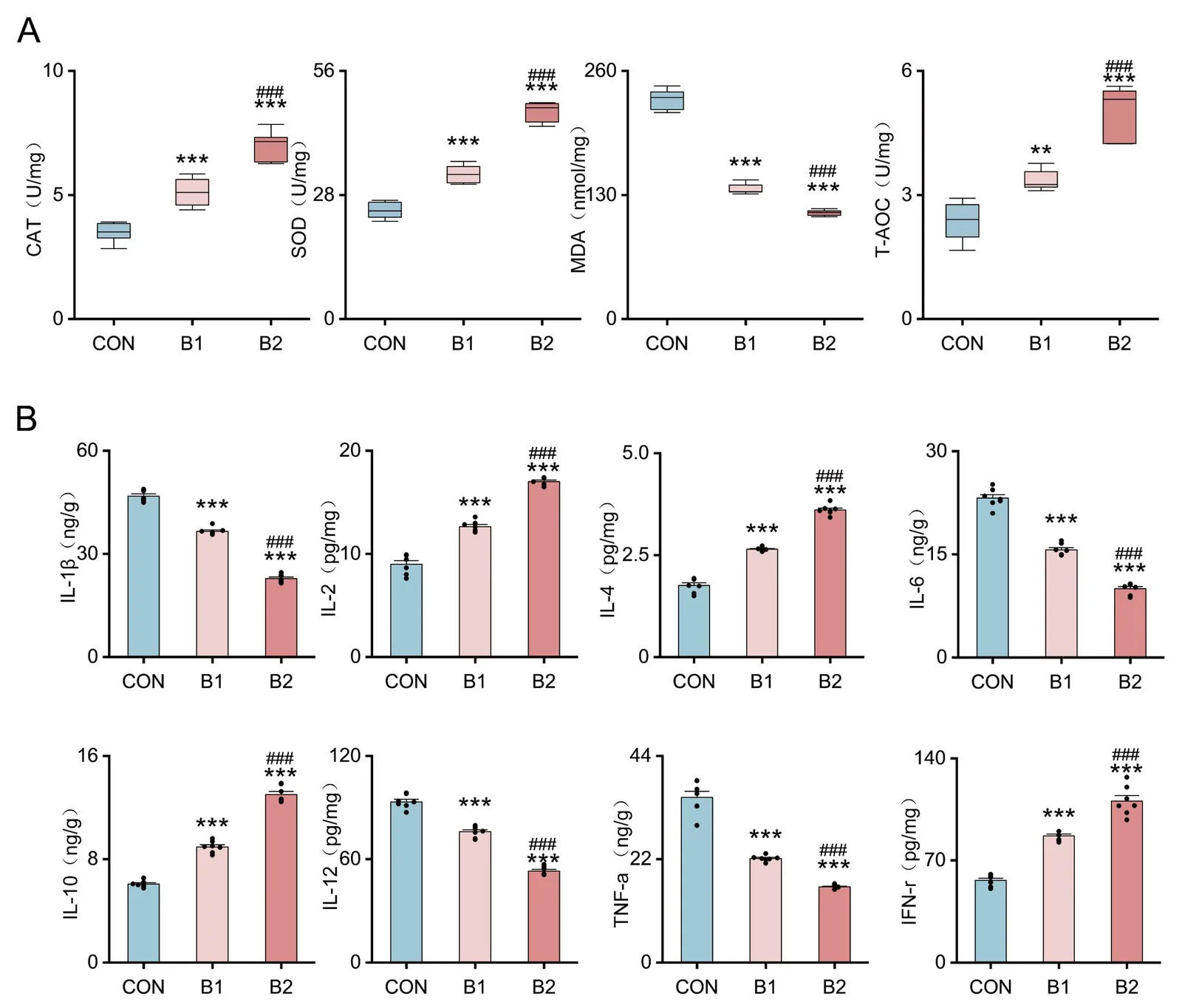
Jejunal antioxidant capacity and inflammatory cytokine levels. (**A**) Jejunal antioxidant-related indicators: catalase (CAT), superoxide dismutase (SOD), malondialdehyde (MDA), and total antioxidant capacity (T-AOC) of different experimental groups; (**B**) jejunal immune-associated cytokines, including pro-inflammatory cytokines (IL-1β, IL-6, IL-12, TNF-α), anti-inflammatory cytokines (IL-4, IL-10), as well as immune regulatory factors (IL-2, IFN-γ) of different experimental groups. CON, basal diet control group without β-carotene; B1, basal diet supplemented with microencapsulated β-carotene; B2, basal diet supplemented with nanoemulsified β-carotene. Asterisks (**, ***): significant difference compared with the CON group at *p* < 0.01 and *p* < 0.001, respectively. Hash symbols (###): significant difference between B1 and B2 groups at *p* < 0.01 and *p* < 0.001, respectively. *n* = 7. Box plots and bar charts display the distribution of individual replicate values.

To systematically evaluate the regulatory effects of the two formulations of β-carotene on the jejunal redox balance and local immune homeostasis in piglets, jejunal antioxidant biomarkers and inflammatory cytokines were determined in this trial, and the results are presented in Figure 7. The results for antioxidant indicators showed that, compared with the CON group, both the B1 and B2 groups significantly increased the activities of jejunal catalase (CAT), superoxide dismutase (SOD) and total antioxidant capacity (T-AOC), while significantly reducing the content of malondialdehyde (MDA), a lipid peroxidation product (*p* < 0.001). Comparison between the B1 and B2 groups indicated that the antioxidant effect of the B2 group was superior to that of the B1 group: the activities of jejunal CAT, SOD and T-AOC were higher, and the MDA content was lower in the B2 group (*p* < 0.001; Figure 7A).

Jejunal inflammatory cytokines exhibited a synergistic and consistent variation trend with the antioxidant indicators. Compared with the CON group, supplementation with either microencapsulated or nanoemulsified β-carotene significantly downregulated the expression of pro-inflammatory mediators IL-1β, IL-6, IL-12 and TNF-α and significantly upregulated anti-inflammatory cytokines IL-4 and IL-10, as well as immune regulatory factors IL-2 and IFN-γ (*p* < 0.001). Significant differences were observed in all immune indicators between the B1 and B2 groups, and the jejunum of piglets in the B2 group showed a more balanced anti-inflammatory phenotype (*p* < 0.001; Figure 7B).

### 3.9. Effects of β-Carotene Forms on Jejunal Microbial Diversity and Community Composition of Piglets

**Figure 8:**
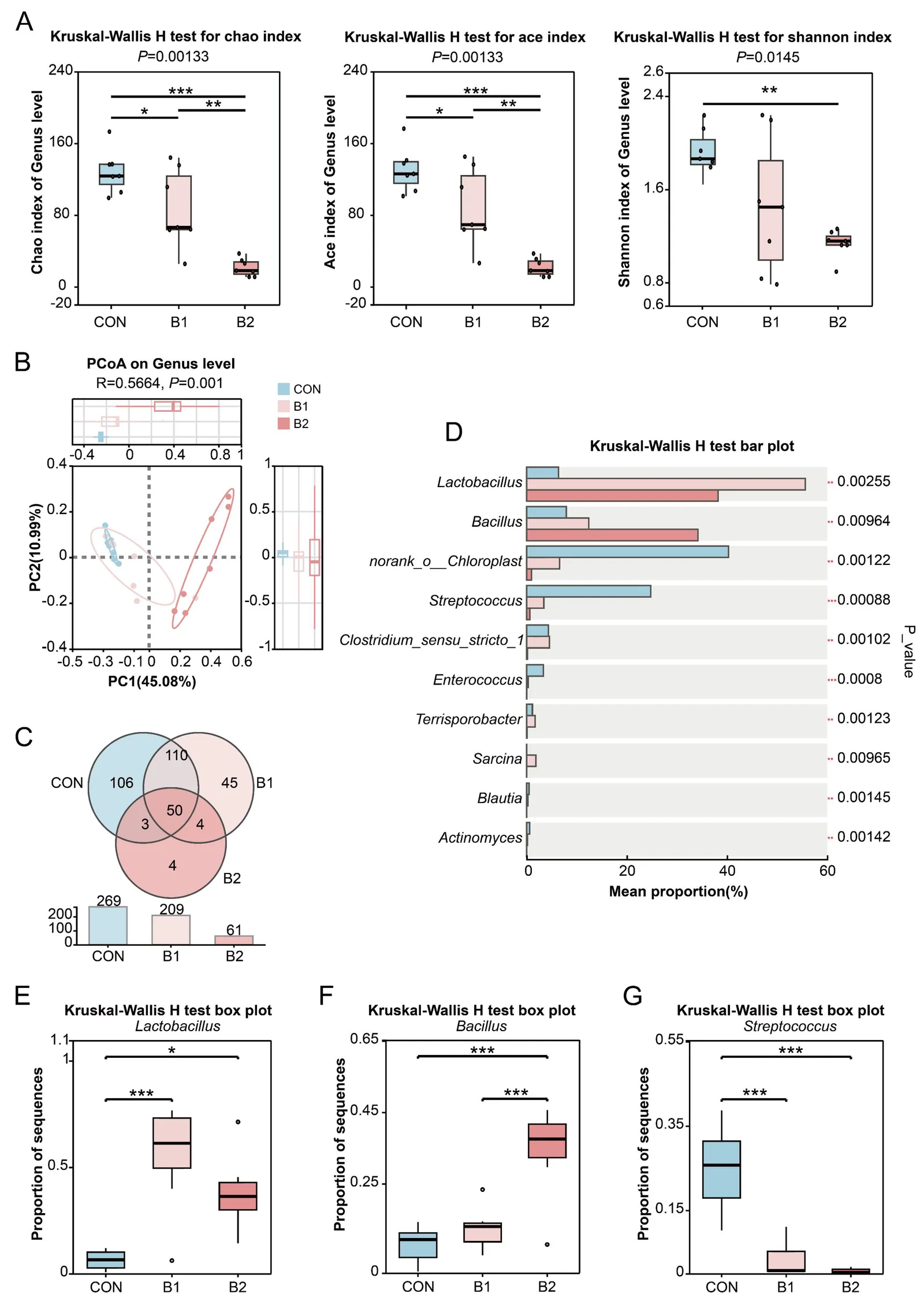
Jejunal microbial diversity and composition profiles. (**A**) Box plots of Chao, Ace and Shannon alpha diversity indices of jejunal microbiota across three groups; intergroup differences were analyzed by Kruskal-Wallis H test. (**B**) Principal coordinate analysis (PCoA) of genus-level microbiota based on Bray–Curtis distance, ANOSIM test: R = 0.5664, *p* = 0.001. (**C**) Venn diagram of genus-level taxa in three groups; the bar chart at the bottom shows the total number of genera detected in each group. (**D**) Bar plot displaying the average relative abundance of 10 significantly differential genera screened by Kruskal–Wallis multiple-group comparison. (**E**–**G**) Box plots of relative abundance of three representative core genera, *Lactobacillus*, *Bacillus* and *Streptococcus*. CON, blank control group; B1, microencapsulated β-carotene group; B2, nanoemulsified β-carotene group. *n* = 7. * *p* < 0.05, ** *p* < 0.01, *** *p* < 0.001.

To explore the microbial mechanism by which different delivery formulations of β-carotene mediate jejunal health in piglets, the genus-level alpha diversity of jejunal microbiota was evaluated using Chao, Ace and Shannon indices in the present study (Figure 8A). The Kruskal–Wallis H test revealed significant overall intergroup differences among the three treatments (Chao and Ace indices: *p* = 0.00133; Shannon index: *p* = 0.0145). All three diversity indices of the CON group were significantly higher than those of the B1 and B2 groups, and the species richness indicators of group B1 were higher than those of group B2. Collectively, these findings illustrated that both microcapsule and nanoemulsion delivery systems of β-carotene could alter the species richness and evenness of jejunal microbial communities in piglets; the advantages and disadvantages of such microbial shifts need to be comprehensively interpreted combined with subsequent multi-dimensional community analysis of microbiota.

Principal coordinate analysis (PCoA) based on the Bray–Curtis distance was conducted for genus-level jejunal microbiota (Figure 8B). The ANOSIM test obtained R = 0.5664 and *p* = 0.001, indicating highly significant overall differences in microbial community composition across the CON, B1 and B2 groups. The clustering ellipses showed that the sample range of the CON group was partially overlapped and covered by the B1 group, reflecting partial similarity between the communities of the two groups. In contrast, samples of the B2 group formed a fully independent cluster without any overlap with CON and B1, demonstrating that the nanoemulsion β-carotene exerted a stronger reshaping effect on the jejunal microbial structure. Venn diagram analysis of species composition (Figure 8C) further revealed the taxonomic basis for the separation of microbial clusters. A total of 50 core genera were shared by all three groups. The CON group contained 106 unique genera, the B1 group had 45 unique genera, while only 4 unique genera were detected in the B2 group. The total number of detected genera followed the gradient: CON (269) > B1 (209) > B2 (61). Taken together, the results of PCoA and Venn diagram indicated that both microcapsule and nanoemulsion formulations of β-carotene could screen and reshape jejunal microbiota in piglets, and nanoemulsified β-carotene exerts a stronger intervention effect and leads to a higher degree of homogenization of the intestinal microbial community.

Kruskal–Wallis multiple-group comparison was conducted based on genus-level relative abundance. The top 10 genera with the most significant intergroup differences were screened and visualized via bar plots to display the average proportions across treatments (Figure 8D). As shown in the figure, the CON group was enriched with abundant conditional pathogenic taxa including *Streptococcus*, *Enterococcus* and *Actinomyces*, all of which exhibited the lowest relative abundance in the B2 group. The B1 group exhibited the prominent enrichment of multiple beneficial genera such as *Lactobacillus*, *Terrisporobacter* and *Clostridium_sensu_stricto_1* while the B2 group was characterized by specific high enrichment of *Bacillus*. Overall, B1 possessed a richer spectrum of beneficial microbiota, whereas B2 displayed a dramatic upregulation of single probiotic *Bacillus*. To intuitively reveal the variation pattern of core microbiota, three representative key genera were selected for separate box plots to further quantify the abundance discrepancies among the three groups (Figure 8E–G). Figure 8E illustrates the relative abundance of *Lactobacillus*, which followed the gradient CON < B2 < B1. Multiple comparisons showed that the abundance of *Lactobacillus* in the B1 group was significantly higher than that in the CON group (*p* < 0.001) and significantly higher than that in the B2 group (*p* < 0.05), indicating that the microcapsule delivery system facilitated the colonization of jejunal *Lactobacillus*. Figure 8F presents the distribution of Bacillus, with an ascending gradient CON < B1 < B2. The B2 group possessed an extremely higher abundance of *Bacillus* compared with the CON and B1 groups (*p* < 0.001), demonstrating that the nanoemulsion formulation could specifically enrich *Bacillus*. Figure 8G shows the relative abundance of the conditional pathogen Streptococcus, which exhibited a descending gradient CON > B1 > B2. Its abundance in the CON group was significantly higher than that in two intervention groups (*p* < 0.001), revealing that both β-carotene delivery formulations effectively suppressed the proliferation of intestinal Streptococcus, with the nanoemulsion (B2 group) performing best.

The microcapsule delivery system exerted regulatory effects mainly by the targeted enrichment of beneficial bacteria such as *Lactobacillus*, while the nanoemulsion delivery system significantly enriched *Bacillus* and drastically reduced the relative abundance of conditional pathogens including *Streptococcus*. This pattern can explain the ranking of the alpha diversity indices (CON > B1 > B2) shown in Figure 8A: the control group possessed the highest microbial diversity but was dominated by conditional pathogens; the B1 group partially suppressed the proliferation of conditional pathogens and enriched beneficial bacteria to remodel intestinal microbial community; the B2 group exhibited the strongest inhibitory effect against conditional pathogens, accompanied by the highest homogenization of jejunal microbiota. Collectively, these findings illustrate the underlying mechanism by which microencapsulated and nanoemulsified β-carotene improve the intestinal health and growth performance of weaned piglets from the perspective of intestinal microorganisms.

### 3.10. Effects of β-Carotene Delivery Forms on Colonic Antioxidant Capacity and Inflammatory Cytokine Profiles of Piglets

**Figure 9:**
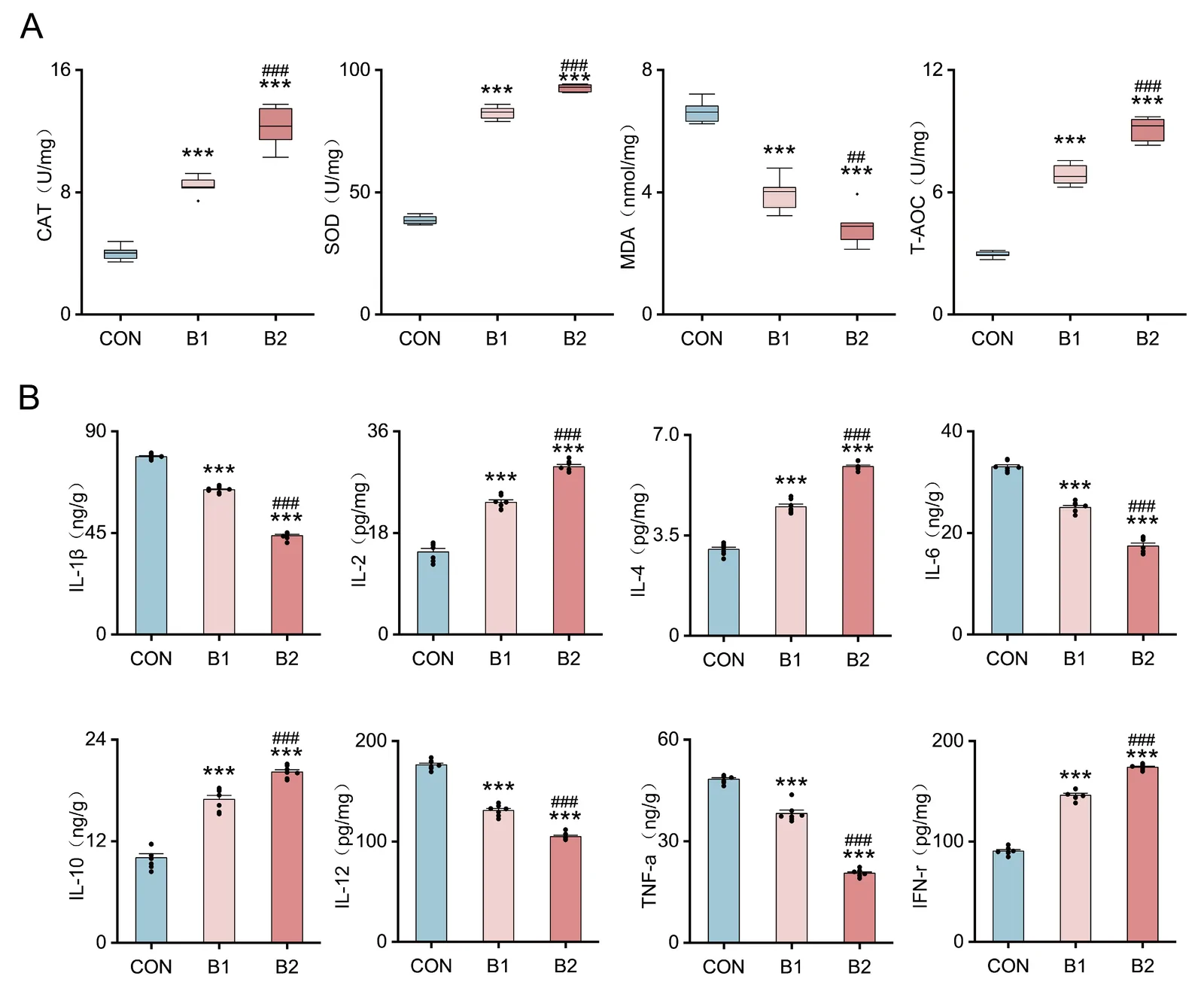
Colonic antioxidant capacity and inflammatory cytokine levels. (**A**) Colonic antioxidant-related indicators: catalase (CAT), superoxide dismutase (SOD), malondialdehyde (MDA), and total antioxidant capacity (T-AOC) of different experimental groups; (**B**) colonic immune-associated cytokines, including pro-inflammatory cytokines (IL-1β, IL-6, IL-12, TNF-α), anti-inflammatory cytokines (IL-4, IL-10), and immune regulatory factors (IL-2, IFN-γ) of different experimental groups. CON, basal diet control group without β-carotene; B1, basal diet supplemented with microencapsulated β-carotene; B2, basal diet supplemented with nanoemulsified β-carotene. Asterisks (***): significant difference compared with the CON group at *p* < 0.001. Double hash symbols (##): significant difference between B1 and B2 groups at significant difference at *p* < 0.01. Hash symbols (###): significant difference between B1 and B2 groups at *p* < 0.01 and *p* < 0.001, respectively. *n* = 7. Box plots and bar charts display the distribution of individual replicate values.

To fully reveal the regulatory effects of different delivery forms of β-carotene on intestinal redox homeostasis and mucosal immunity throughout the intestinal tract of piglets, we further determined the antioxidant indicators and inflammatory cytokines in colonic tissues, and the results are shown in Figure 9. Compared with the CON group, both the B1 and B2 groups significantly increased the activities of colonic CAT, SOD and T-AOC and reduced the content of the lipid peroxidation product MDA (*p* < 0.001). Intergroup comparison revealed that the B2 group achieved a remarkably better antioxidant improvement effect in the colon than the B1 group, with higher CAT, SOD and T-AOC activities and lower MDA levels (*p* < 0.001). The changing trends of colonic inflammatory cytokines were consistent with those of antioxidant indicators. Compared with the CON group, both groups of β-carotene significantly downregulated pro-inflammatory cytokines IL-1β, IL-6, IL-12 and TNF-α, while simultaneously upregulating anti-inflammatory cytokines IL-4, IL-10 as well as immune regulatory factors IL-2 and IFN-γ (*p* < 0.001). Significant differences in all immune-related indicators were detected between the B1 and B2 groups, and the B2 group maintained a more balanced profile of pro- and anti-inflammatory cytokines in the colon (*p* < 0.001).

## 4. Discussion

As a natural antioxidant, β-carotene can scavenge intracellular reactive oxygen species, alleviate oxidative-stress-induced damage, suppress oxidative-stress-mediated inflammatory responses, and modulate the proliferation and differentiation of immune cells, thereby regulating host immune function [19]. Nevertheless, its poor water-solubility and low bioavailability severely limit its practical application. In the present study, two encapsulated delivery systems, namely microencapsulated β-carotene (Group B1) and nanoemulsified β-carotene (Group B2), were adopted to systematically evaluate their effects on growth performance, antioxidant capacity, inflammatory status, intestinal barrier integrity, and jejunal microbial composition in weaned piglets. The results demonstrated that both the B1 and B2 treatments significantly improved all the above-mentioned parameters. Moreover, the nanoemulsion formulation (B2) exerted markedly superior effects over the microcapsule formulation for nearly all detected indices. These findings highlight the critical role of delivery-system construction in modulating the in vivo biological activity of β-carotene.

The superior biological performance of the nanoemulsion formulation compared with microcapsules can be fundamentally attributed to their distinct physicochemical properties and in vivo absorption-related behaviors. Although both encapsulation systems offered comparable protection against the lipid oxidation of β-carotene under accelerated storage conditions (Figure 1D), the nanoscale droplet characteristics of B2 nanoemulsion endow it with unique advantages during gastrointestinal transit and absorption. The size of 1280–1300 nm corresponds to oil-core droplets released upon hydration of the microcapsule powder, simulating the physiological status of microcapsules when exposed to aqueous gastrointestinal fluids in vivo. These relatively large released oil droplets tend to be trapped within the intestinal mucus mesh and struggle to penetrate the mucus layer. In contrast, nanoemulsions with a particle size of 110–140 nm can efficiently penetrate intestinal mucus and be internalized by intestinal epithelial cells via multiple pathways. Such differences in mucus-penetrating and cellular-uptake efficiency could partially explain the markedly elevated plasma retinol concentration observed in B2 piglets (Figure 2). Plasma retinol, instead of intact β-carotene, was chosen to assess the β-carotene bioavailability in the present study. In pigs, the majority of absorbed β-carotene undergoes conversion into retinol in intestinal epithelial cells before entering blood circulation, while only a minor proportion of intact β-carotene remains untransformed. Hence, plasma retinol serves as a more suitable indicator to characterize the in vivo utilization of provitamin A. The significantly higher plasma retinol level in Group B2 indicates that nanoemulsification can effectively improve the in vivo bioavailability of β-carotene, which accounts for why the nanoemulsion formulation outperforms microcapsules in antioxidant, anti-inflammatory and growth-promoting effects. To disentangle the contributions of formulation property and feed-intake discrepancy, an auxiliary mouse gavage experiment was conducted. Mice in group B1 received microencapsulated β-carotene fully dispersed in deionized water by gavage, whereas mice in group B2 were administered nanoemulsified β-carotene. The daily gavage dosage of β-carotene was fixed at 20 mg kg^−1^ d^−1^ for both groups, and all other experimental conditions were kept identical. Higher plasma retinol concentration was observed in B2 mice compared with B1 counterparts (unpublished data), which was consistent with the trend shown in Figure 2 of the piglet trial. Collectively, these findings indicate that the elevated feed intake and resultant higher actual β-carotene consumption in B2 piglets are positive biological consequences triggered by the nanoemulsified delivery system. The difference in bioavailability derived from varied delivery forms, rather than intake discrepancy, constitutes the fundamental reason for the divergent growth performance between B1 and B2 piglets.

As a lipophilic carotenoid, β-carotene scavenges reactive oxygen species, inhibits lipid peroxidation [20], and modulates lipid metabolism in tissue cells [21]. Consistent patterns of antioxidant and inflammatory-related indices were observed across the three examined tissues (liver, jejunum and colon). Dietary supplementation with microencapsulated and nanoemulsified β-carotene significantly increased the activities of catalase, superoxide dismutase and total antioxidant capacity in tissues, while reducing the malondialdehyde levels. These results suggest that both encapsulated formulations effectively mitigate lipid peroxidation damage and reinforce the endogenous antioxidant defense system. This observation is consistent with the well-recognized free-radical-scavenging capacity of β-carotene; its long conjugated polyene chain can quench singlet oxygen and neutralize peroxyl radicals, thereby alleviating oxidative-stress-induced injury. Meanwhile, both B1 and B2 treatments substantially remodeled the intestinal immune microenvironment, downregulated the expression of pro-inflammatory cytokines (IL-1β, IL-6, IL-12 and TNF-α), and upregulated anti-inflammatory cytokines (IL-4, IL-10), as well as immune regulatory factors (IL-2, IFN-γ). This immunomodulatory effect is mainly ascribed to retinoic acid, an active in vivo metabolite of β-carotene. Retinoic acid can attenuate tissue inflammation by suppressing oxidative-stress-triggered inflammatory pathways, and it directly governs the differentiation and polarization of immune cells such as macrophages and T lymphocytes, so as to restrain excessive inflammatory responses [22] and maintain intestinal immune homeostasis [23]. Weaned piglets are susceptible to oxidative stress and chronic low-grade inflammation, which impair the intestinal barrier function, hamper nutrient digestion and absorption [24], and ultimately depress growth performance [25]. Owing to its intrinsic free-radical-scavenging activity, β-carotene can mitigate intestinal oxidative damage and modulate cytokine secretion to relieve mucosal inflammation [26] and improve the growth performance of animals during early growth and development [27]. In addition, the two β-carotene delivery systems can improve the growth performance of weaned piglets via elevating digestive enzyme activities. Increased activities of amylase and lipase in the jejunum of piglets in groups B1 and B2 accelerate the degradation of dietary starch and fat, thereby facilitating the digestion and absorption of nutrients. These are in line with the favorable growth performance as well as antioxidant and anti-inflammatory effects in the jejunum and colon observed in Groups B1 and B2.

Intestinal morphological integrity serves as a reliable indicator for digestive and absorptive capacity. The increased villus height and crypt depth represent an enlarged intestinal absorptive area and the accelerated renewal of intestinal epithelial cells, which are core indices for evaluating intact intestinal development and sound digestive-absorptive function [28]. Weaning readily triggers intestinal oxidative-stress injury in piglets [29] disrupts the villus–crypt structure and impairs nutrient absorption. Intestinal oxidative stress and low-grade inflammation can compromise mucosal integrity, suppress digestive-enzyme secretion and hinder nutrient uptake [30]. In the present study, dietary supplementation with either B1 or B2 significantly increased the villus height and crypt depth in the duodenum, jejunum and ileum, with the most pronounced restorative effects detected in Group B2. This is physiologically meaningful: taller villi provide a larger absorptive surface area, whereas deeper crypts reflect enhanced epithelial renewal capacity. The improved morphological structure was functionally confirmed by reduced serum diamine oxidase and D-lactate levels in Groups B1 and B2. Serum diamine oxidase and D-lactate are two classic circulating biomarkers for assessing intestinal mucosal permeability [31]. Diamine oxidase is a specific intracellular enzyme predominantly located in small-intestinal villus epithelial cells; once intestinal barrier damage occurs, and epithelial integrity is disrupted, diamine oxidase leaks into the bloodstream and elevates serum diamine–oxidase activity [32]. Similarly, D-lactate is a metabolite exclusively produced by intestinal microbiota, which can enter systemic circulation only through impaired intestinal tight junctions [33]. Accordingly, lower serum concentrations of diamine oxidase and D-lactate indicate tighter intestinal epithelial junctions and superior intestinal-barrier integrity [34]. Piglets in Group B2 exhibited the lowest serum diamine oxidase and D-lactate levels on both day 14 and day 28, demonstrating that the nanoemulsion formulation afforded the most robust protection against weaning-provoked barrier disruption.

The intestine acts as a vital barrier defending the host against toxins and exogenous antigens, and alterations in its microbiota can directly shape host intestinal immune and oxidative-stress status. The jejunum constitutes a key site for nutrient absorption, and the present study revealed distinctly different jejunal microbiota-modulating patterns induced by the two β-carotene delivery systems. As shown in the results, the CON group was enriched with abundant opportunistic pathogenic taxa, including *Streptococcus* [35], *Enterococcus* [36] and *Actinomyces* [37], whose relative abundances reached the lowest values in Group B2. Group B1 displayed a significant enrichment of multiple beneficial genera such as *Lactobacillus* [38], *Sporobacter* [39] and *Clostridium_sensu_stricto* [40], whereas Group B2 was characterized by a specific high enrichment of *Bacillus* [41]. Such differentiated microbial profiles may be associated with their divergent intestinal-release behaviors and modes of action; different encapsulation carriers can alter the intestinal-exposure profiles of bioactive compounds and thus mediate distinct microbial responses [42]. These findings suggest that remodeling the jejunal microbial community is one critical pathway by which encapsulated β-carotene modulates intestinal health in weaned piglets.

## 5. Conclusions

Nanoemulsified β-carotene exerts superior antioxidant, anti-inflammatory and growth-promoting effects compared with microencapsulated β-carotene. This may be attributed to its improved absorption efficiency, higher bioavailability, and potent modulatory effects on intestinal microbiota. These findings provide a mechanistic framework for understanding how delivery-system design governs the in vivo efficacy of lipophilic bioactive compounds. From a practical perspective, the nanoemulsion strategy represents a promising approach to improve the application efficiency of β-carotene in animal production.

## Figures and Tables

**Table 1:** Ingredients’ composition and nutritive levels of the control diet for the piglets.

Item, %	CON	B1	B2
Maize	55.55	55.55	55.55
Soybean meal	15.70	15.70	15.70
Extruded soybean	5.00	5.00	5.00
Soy protein concentrate	4.00	4.00	4.00
Fish meal	4.00	4.00	4.00
Low-protein whey powder	8.00	8.00	8.00
Sucrose	3.00	3.00	3.00
Soy oil	1.30	1.30	1.30
Dicalcium phosphate	1.20	1.20	1.20
Limestone	0.50	0.50	0.50
NaCl	0.30	0.30	0.30
L-Lysine-HCl	0.30	0.30	0.30
DL-Methionine	0.20	0.20	0.20
L-Threonine	0.15	0.15	0.15
L-Tryptophan	0.10	0.10	0.10
L-Valine	0.20	0.20	0.20
Vitamins and trace minerals ^1^	0.50	0.50	0.50
Total	100.00	100.00	100.00
Nutritive level ^2^			
Digestible energy, MJ/kg	14.50	14.50	14.50
Crude protein, %	18.50	18.50	18.50
Calcium, %	0.80	0.80	0.80
Phosphorus, %	0.60	0.60	0.60
β-carotene, mg/kg ^3^	12.65 ± 0.18	264.44 ± 4.35	263.31 ± 5.16

^1^ Premix provided the following per kilogram of feed: vitamin A, 5000 IU; vitamin D3, 2000 IU; vitamin E, 30 IU; vitamin K3, 3 mg; vitamin B1, 2.5 mg; vitamin B2, 4.0 mg; vitamin B6, 3.0 mg; vitamin B12, 12 μg; nicotinic acid, 40 mg; thiamine, 3 mg; riboflavin, 6 mg; D-pantothenic acid, 15 mg; folic acid, 1.2 mg; biotin, 50 μg; Fe, 90.0 mg; Zn, 75.0 mg; Mn, 40.0 mg; I, 0.35 mg; Se, 0.3 mg. ^2^ Nutritive levels were calculated values on an air-dry basis. ^3^ Values represent the measured β-carotene concentrations in experimental diets. No exogenous β-carotene was supplemented in the CON group, and its β-carotene content originated intrinsically from feed ingredients. The β-carotene contents of B1 diet and B2 diet were determined after thorough mixing of β-carotene microcapsule preparation and β-carotene nanoemulsion with basal diet, respectively.

**Table 2:** Effects of dietary supplementation with β-carotene of different delivery forms on growth performance of piglets over a 28-day feeding trial.

Items	CON ^1^	B1	B2	*p* Value
Initial weight (kg)	7.75 ± 1.05	7.74 ± 1.02	7.75 ± 1.08	0.3684
Final weight (kg)	27.89 ± 0.34 ^a^	28.94 ± 0.47 ^b^	30.80 ± 0.41 ^c^	<0.0001
Average daily gain (g)	719.35 ± 24.08 ^a^	756.99 ± 12.99 ^b^	823.07 ± 21.75 ^c^	<0.0001
Average daily feed intake (g)	1172.52 ± 35.29 ^a^	1209.65 ± 21.64 ^b^	1282.51 ± 24.87 ^c^	<0.0001
Feed intake/Weight gain	1.63 ± 0.07	1.60 ± 0.03	1.56 ± 0.04	0.0583
Daily β-carotene intake (mg/d) ^2^	14.83 ± 2.11 ^a^	319.88 ± 17.26 ^b^	337.70 ± 19.09 ^b^	—

^1^ CON, basal diet; B1, basal diet supplemented with microencapsulated β-carotene; B2, basal diet supplemented with nanoemulsified β-carotene. Values are means ± standard deviation. Means within the same row with different superscript letters differ significantly (*p* < 0.05). ^2^ Daily β-carotene intake was a calculated value derived from average daily feed intake and analyzed β-carotene concentration in diets, instead of direct measured data.

## Data Availability

The data presented in this study are available on request from the corresponding author due to privacy.

## Abbreviations

CON	Control group
B1	Microencapsulated β-carotene treatment group
B2	Nanoemulsified β-carotene treatment group
